# The role of a second diffusing component on the Gill–Rees stability problem

**DOI:** 10.1038/s41598-022-20966-2

**Published:** 2022-11-11

**Authors:** B. M. Shankar, K. V. Nagamani, I. S. Shivakumara

**Affiliations:** 1 Department of Mathematics, PES University, Bangalore, 560 085 India; 2 Department of Mathematics, Bangalore University, Bangalore, 560 056 India

**Keywords:** Applied mathematics, Mechanical engineering

## Abstract

The stability of natural convection in a vertical porous layer using a local thermal nonequilibrium model was first studied by Rees (Transp Porous Med 87:459–464, 2011) following the proof of Gill (J Fluid Mech 35:545–547, 1969), called the Gill–Rees stability problem. The aim of the present study is to investigate the implication of an additional solute concentration field on the Gill–Rees problem. The stability eigenvalue problem is solved numerically and some novel results not observed in the studies of double-diffusive natural convection in vertical porous (local thermal equilibrium case) and non-porous layers are disclosed. The possibility of natural convection parallel flow in the basic state becoming unstable due to the addition of an extra diffusing component is established. In some cases, the neutral stability curves of stationary and travelling-wave modes are connected to form a loop within which the flow is unstable indicating the requirement of two thermal Darcy–Rayleigh numbers to specify the stability/instability criteria. Moreover, the change in the mode of instability is recognized in some parametric space. The results for the extreme cases of the scaled interphase heat transfer coefficient are discussed.

## Introduction

The stability of natural convection arising due to thermal buoyancy in a porous medium has received considerable attention because of its relevance in various applications such as geophysics, building industry, post-accident heat removal from pebble-bed reactors, modelling of convection in the underground storage of CO_2_, the intensification of heat transfer in compact heat exchangers where metal foams are used. The study has remained a subject of active research and the growing volume of work in this area is amply documented in the books by Straughan^1^, Nield and Bejan^2^ and Barletta^3^.

In his seminal paper, Gill^4^ proposed rigorous mathematical proof to establish the absence of thermoconvective instability in a differentially heated vertical layer of Darcy porous medium. Other authors further enriched Gill’s results by taking into account several aspects such as the inclusion of time derivative term in the momentum equation^5^, non-Newtonian fluid behavior^6,7^, permeable boundaries^8^, Brinkman term^9,10^ and the horizontal heterogeneity in permeability^11^. Among these authors, Rees^5^, and Barletta and Alves^6^ showed that the natural convection parallel flow in the basic state remains stable as propounded by Gill^4^. Whereas, Shankar and Shivakumara^7,11^, Barletta^8^ and Shankar et al.^9,10^ established that the instability of base flow emerges at sufficiently large Darcy–Rayleigh numbers. The usual assumption made in all these studies is that the local thermal equilibrium (LTE) prevails between the solid and fluid phases of the porous medium.

It is expected that LTE will be broken down and the temperatures of solid as well as fluid phases may be no longer identical under highly unsteady conditions, or when the differences between the thermal conductivities of the two phases exist^2^. In such circumstances, one has to utilize the local thermal nonequilibrium (LTNE) model in which two temperature equations, one for the fluid phase and another for the solid phase, are considered with a coupling term in both the equations describing the interphase heat transfer. One of the key issues in dealing with LTNE model is the estimation of the interphase heat transfer coefficient and Rees^12,13^ has scrutinized this problem for various classes of materials. The condition of LTE is approached when the coefficient of interphase heat transfer assumes large values. From an application point of view, LTNE model is playing an important role in porous media, such as computer chips via the use of porous metal foams^14,15^, drying/freezing of foods^16,17^, microwave heating^18^ etc. Rees^19^ was the first to extend the work of Gill^4^ to account LTNE effects and showed that the flow remains stable for all infinitesimal perturbations (hereafter we refer to it as the Gill–Rees stability problem). Scott and Straughan^20^ were able to prove that a nonlinear stability analysis, based on the energy method, leads to the same response declared by Gill^1^. Later, Celli et al.^21^ reconsidered the analysis carried out by Rees^19^ on altering the velocity boundary conditions from the impermeable to permeable and showed numerically the possibility of base flow becoming unstable. Further developments on the Gill–Rees problem were taken up by considering viscoelastic effects^22^, uniform internal heat generation in both the phases of the porous medium^23^ and the combined effects of Darcy–Prandtl number as well as the density maximum property^24^. It was established that instability occurs in all these cases under certain conditions.

In some practical problems encountered in nature and in the industry, such as in devising an effective method of disposing of waste material and extraction of energy, some dissolved substance may exist in addition to temperature contributing in opposite senses to the buoyancy gradient.The instability occurring due to this process is known as thermosolutal convection or double-diffusive convection. The comprehensive literature existing on double-diffusive convection in porous media is well documented in the books by Straughan^25^ and Nield and Bejan^2^. Nonetheless, the stability of double-diffusive natural convection in a vertical porous layer has not received due attention in the literature and it is in the much-to-be desired state. Inspired by the pioneering paper by Gershuni et al.^26^, a few authors have analyzed the stability of convective flow of a binary mixture in a vertical porous layer and, in particular, this work was examined for all values of the Darcy–salinity Rayleigh number by Khan and Zebib^27^. It was found that for salinity Rayleigh number $R_{S} < 7.901$ there are no two-dimensional instabilities, however, instability sets in via stationary mode for $R_{S} \ge 7.901$. The stability of natural convection induced by buoyancy due to temperature and solute concentration fields in a vertical porous layer was investigated in detail by Shankar et al.^28^. It was observed that there exists a solute Darcy–Rayleigh number space within which the flow gets destabilized and beyond which it stabilizes reversely depending on the values of the Lewis number. The above studies were mainly based on the assumption that the fluid and porous-medium phases are everywhere in LTE.

As propounded by the previous studies, the consideration of LTNE model in the study of heat and mass transfer in a porous medium is of paramount importance. In a porous medium, heat is shared between the fluid and the solid skeleton and thus the process is double-advective while the solute concentration is confined only to the pore space. Moreover, the thermal signals are advected more slowly than solute concentration signals, and this difference in advection rates may be crucial to the stability aspects of the system^29^. The aim of this paper is to explore the influence of an additional diffusing component on the Gill–Rees stability problem. More precisely, the stability of thermosolutal convection in a vertical porous layer is studied numerically using a LTNE model. The two end vertical boundaries are assumed to be impermeable and maintained at unequal uniform temperatures and solute concentrations. Within this more general scheme, the prediction of instability is validated through a stability eigenvalue problem derived by adopting a modal analysis. The effect of additional diffusing component is discussed for the neutral stability curves and the critical values of the wavenumber and of the thermal Darcy–Rayleigh number.

## Statement of the problem and governing equations

**Figure 1:**
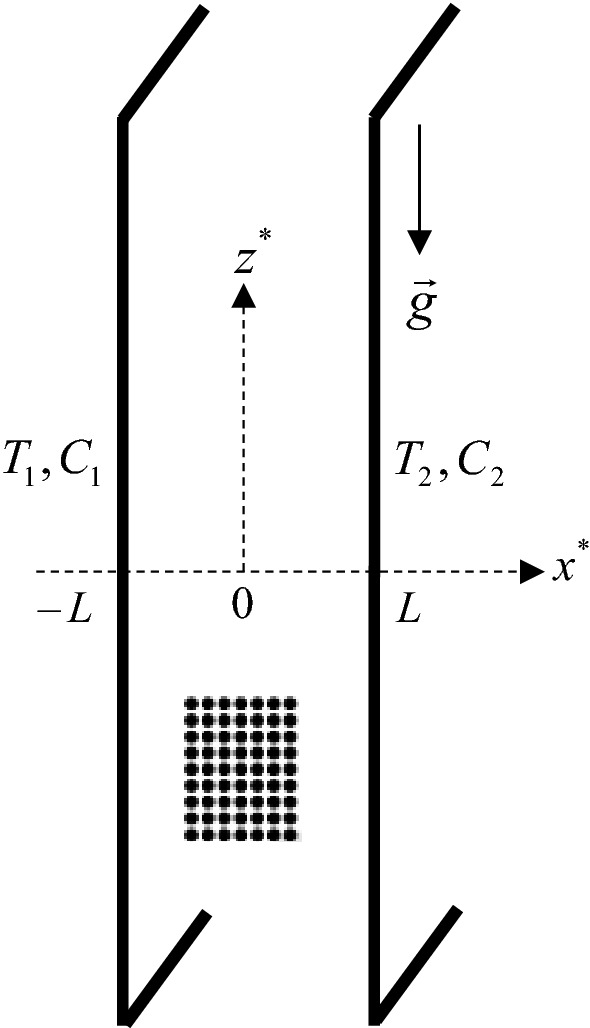
A sketch of the vertical porous layer.

A vertical porous layer of thickness $2L$ is considered wherein the two end vertical impermeable walls are maintained at uniform but different temperatures $T_{1}$ and $T_{2}$, with $T_{2} > T_{1}$ and solute concentrations $C_{1}$ and $C_{2}$, with $C_{2} > C_{1}$. A Cartesian reference frame $(x^{*} ,y^{*} ,z^{*} )$ is chosen so that the $x^{*}$-axis is horizontal and perpendicular, $y^{*}$-axis is horizontal and parallel, while the $z^{*}$-axis is vertical and parallel to the porous channel. The origin of the axes is in the middle of the porous layer and the acceleration due to gravity $\vec{g} = - g\hat{k}$, where $\hat{k}$ is the unit vector in the vertical $z^{*}$-direction. A sketch of the porous channel cross-section in the $x^{*} z^{*}$-plane is shown in Fig. 1. The porous medium is considered to be homogeneous, isotropic and the LTNE model is invoked with two temperature equations, one for the fluid phase and another for the solid phase. The fluid density $\rho_{f}$ varies linearly with fluid temperature $T_{f}^{*}$ and the solute concentration $C^{*}$ of the dissolved species in the form

1 $$\rho_{f} \left( {T_{f}^{ * } ,C^{*} } \right) = \rho_{0} \left\{ {1 - \beta_{T} \left( {T_{f}^{ * } - T_{0} } \right) + \beta_{S} \left( {C^{*} - C_{0} } \right)} \right\},$$

where $\beta_{T}$ is the volumetric thermal expansion coefficient, $\beta_{S}$ is the solute expansion coefficient, $T_{0} = (T_{1} + T_{2} )/2$ is the reference temperature, $C_{0} = (C_{1} + C_{2} )/2$ is the reference solute concentration and $\rho_{0} = \rho_{f} \left( {T_{0} ,C_{0} } \right)$. The starred symbols denote the dimensional variables. The dimensionless dependent variables $\left( {\vec{q},T_{f} ,T_{s} ,C,P} \right)$, space coordinates $(x,y,z)$ and time $t$ are defined as follows:

2 $$\vec{q}^{ * } \frac{L}{{\varepsilon \kappa_{f} }} = \vec{q},\frac{{\left( {T_{f,s}^{ * } - T_{0} } \right)}}{{\left( {T_{2} - T_{1} } \right)}} = T_{f,s} ,\frac{{\left( {C^{*} - C_{0} } \right)}}{{\left( {C_{2} - C_{1} } \right)}} = C,P^{*} \frac{K}{{\varepsilon \kappa_{f} \mu }} = P,\left( {x^{ * } ,y^{ * } ,z^{ * } } \right)\frac{1}{L} = \left( {x,y,z} \right),t^{*} \frac{{\kappa_{f} }}{{L^{2} }} = t,$$

where $\kappa_{f} = {{k_{f} } \mathord{\left/ {\vphantom {{k_{f} } {(\rho c)_{f} }}} \right. \kern-\nulldelimiterspace} {(\rho c)_{f} }}$, $k_{f}$, $(\rho c)_{f}$, $\vec{q} = (u,v,w),\,\,P,\,\,\varepsilon ,\,\,K,\,\,\mu$ and $T_{s}$ are the effective thermal diffusivity of the fluid, thermal conductivity of the fluid, heat capacity of the fluid, seepage velocity vector, dynamic pressure, porosity, permeability, fluid viscosity and temperature of the solid phase, respectively. Since two-dimensional motion is more unstable than three-dimensional, the stream function $\psi (x,z,t)$ is introduced through $u = - \partial \psi /\partial z$ and $w = \partial \psi /\partial x$. Thus, the dimensionless governing equations under the Oberbeck–Boussinesq approximation and the relevant boundary conditions are Rees^19^ and Shankar et al.^28^

3 $$\frac{{\partial^{2} \psi }}{{\partial x^{2} }} + \frac{{\partial^{2} \psi }}{{\partial z^{2} }} = R_{D} \frac{{\partial T_{f} }}{\partial x} - R_{S} \frac{\partial C}{{\partial x}},$$

4 $$\frac{{\partial T_{f} }}{\partial t} + \frac{\partial \psi }{{\partial x}}\frac{{\partial T_{f} }}{\partial z} - \frac{\partial \psi }{{\partial z}}\frac{{\partial T_{f} }}{\partial x} = \frac{{\partial^{2} T_{f} }}{{\partial x^{2} }} + \frac{{\partial^{2} T_{f} }}{{\partial z^{2} }} + H\left( {T_{s} - T_{f} } \right),$$

5 $$\alpha \frac{{\partial T_{s} }}{\partial t} = \frac{{\partial^{2} T_{s} }}{{\partial x^{2} }} + \frac{{\partial^{2} T_{s} }}{{\partial z^{2} }} - H\gamma \left( {T_{s} - T_{f} } \right),$$

6 $$\frac{\partial C}{{\partial t}} + \frac{\partial \psi }{{\partial x}}\frac{\partial C}{{\partial z}} - \frac{\partial \psi }{{\partial z}}\frac{\partial C}{{\partial x}} = \frac{1}{Le}\left( {\frac{{\partial^{2} C}}{{\partial x^{2} }} + \frac{{\partial^{2} C}}{{\partial z^{2} }}} \right),$$

7 $$\psi = 0\,{\text{at}}\,x = \pm 1,\,T_{f} = T_{s} = C = - 1/2\,{\text{at}}\,x = - 1,\,T_{f} = T_{s} = C = 1/2\,{\text{at}}\,x = 1.$$

In the above equations, $R_{D} = {{\rho_{0} g\beta_{T} (T_{2} - T_{1} )KL} \mathord{\left/ {\vphantom {{\rho_{0} g\beta_{T} (T_{2} - T_{1} )KL} {\varepsilon \mu \kappa_{f} }}} \right. \kern-\nulldelimiterspace} {\varepsilon \mu \kappa_{f} }}$ is the thermal Darcy–Rayleigh number, $R_{S} = {{\rho_{0} g\beta_{s} (C_{2} - C_{1} )KL} \mathord{\left/ {\vphantom {{\rho_{0} g\beta_{s} (C_{2} - C_{1} )KL} {\varepsilon \mu \kappa_{f} }}} \right. \kern-\nulldelimiterspace} {\varepsilon \mu \kappa_{f} }}$ is the solutal Darcy–Rayleigh number, $H = {{hL^{2} } \mathord{\left/ {\vphantom {{hL^{2} } {\varepsilon k_{f} }}} \right. \kern-\nulldelimiterspace} {\varepsilon k_{f} }}$ is the scaled interphase heat transfer coefficient, $\gamma = {{\varepsilon k_{f} } \mathord{\left/ {\vphantom {{\varepsilon k_{f} } {(1 - }}} \right. \kern-\nulldelimiterspace} {(1 - }}\varepsilon )k_{s}$ is the ratio of porosity-modified thermal conductivities, $\alpha = {{(\rho c)_{s} k_{f} } \mathord{\left/ {\vphantom {{(\rho c)_{s} k_{f} } {(\rho c)_{f} }}} \right. \kern-\nulldelimiterspace} {(\rho c)_{f} }}k_{s} = \kappa_{f} /\kappa_{s}$ is the ratio of thermal to solid diffusivities and $Le = {{\kappa_{f} } \mathord{\left/ {\vphantom {{\kappa_{f} } {\kappa_{c} }}} \right. \kern-\nulldelimiterspace} {\kappa_{c} }}$ is the Lewis number, while $h$ is the interphase heat transfer coefficient, $k_{s}$ is the thermal conductivity of the solid, $(\rho c)_{s}$ is the heat capacity of the solid and $\kappa_{c}$ is the solute concentration diffusivity.

## Base flow

A basic flow solution driven by both thermal and solutal buoyancy forces is obtained in a stationary regime with $\psi = \psi_{b} (x)\,,\,\,T_{f} = T_{fb} (x)$, $T_{s} = T_{sb} (x)$ and $C = C_{b} (x)$ in the form

8 $$\psi_{b} (x) = \left( {{1 \mathord{\left/ {\vphantom {1 4}} \right. \kern-\nulldelimiterspace} 4}} \right)\left( {R_{D} - R_{S} } \right)(x^{2} - 1),T_{fb} (x) = T_{sb} (x) = C_{b} (x) = {x \mathord{\left/ {\vphantom {x 2}} \right. \kern-\nulldelimiterspace} 2}.$$

## Linear stability analysis

Following the usual approach of the linearized theory of hydrodynamic stability, perturbations are introduced on the basic state in the form

9 $$\psi = \psi_{b} (x) + \hat{\psi }(x,z,t),T_{f} = T_{fb} (x) + \hat{T}_{f} (x,z,t),T_{s} = T_{sb} (x) + \hat{T}_{s} (x,z,t),C = C_{b} (x) + \hat{C}(x,z,t),$$

where the hat above a quantity designates the perturbation field. Substituting Eq. (9) into the above governing equations, neglecting the nonlinear terms and seeking the solution via the standard Fourier decomposition

10 $$\left( {\psi ,T_{f} ,T_{s} ,C} \right) = \left[ {\Psi ,\Theta ,\Phi ,\Gamma } \right]e^{ia(z - ct)} ,$$

where the quantities $\Psi ,\,\Theta ,\Phi$ and $\Gamma$ are the complex amplitude functions of $x$, $a$ is the wave number in the $z$-direction and $c( = c_{r} + ic_{i} )$ is the complex wave speed, we obtain the disturbance stability equations in the form

11 $$\left( {D^{2} - a^{2} } \right)\Psi - R_{D} D\Theta + R_{S} D\Gamma = 0,$$

12 $$- \left( {{{ia} \mathord{\left/ {\vphantom {{ia} 2}} \right. \kern-\nulldelimiterspace} 2}} \right)\left\{ {\left( {R_{D} - R_{S} } \right)x\Theta - \Psi } \right\} + \left( {D^{2} - a^{2} } \right)\Theta + H\left( {\Phi - \Theta } \right) = - iac\,\Theta ,$$

13 $$\left( {D^{2} - a^{2} } \right)\Phi - H\gamma \left( {\Phi - \Theta } \right) = - iac\,\alpha \,\Phi ,$$

14 $$- \left( {{{ia} \mathord{\left/ {\vphantom {{ia} 2}} \right. \kern-\nulldelimiterspace} 2}} \right)\left\{ {\left( {R_{D} - R_{S} } \right)x\Gamma - \Psi } \right\} + \frac{1}{Le}\left( {D^{2} - a^{2} } \right)\Gamma = - iac\,\Gamma ,$$

where $D$ denotes differentiation with respect to $x$. We note that the flow is stable or unstable for $c_{i} < 0$ or $c_{i} > 0$ and neutrally stable for $c_{i} = 0$.

The associated boundary conditions are

15 $$\,\Psi = \Phi = \Theta = \Gamma = 0\,{\text{at}}\,x = \pm 1.$$

## Gill–Rees growth rate analysis

In this section, we attempt to determine the sign of the growth rate $c_{i}$ using the integral method employed by Gill^4^ and Rees^19^. First, we operate $(D^{2} - a^{2} )$ on Eqs. (12), (13) and (14) and make use of Eq. (11) to eliminate $\Psi$, and get

16 $$\begin{aligned} & \left( {\Theta^{\prime \prime \prime \prime } + a^{4} \Theta - 2a^{2} \Theta^{\prime \prime } } \right) + H\left( {\Phi^{\prime \prime } - a^{2} \Phi } \right) - H\left( {\Theta^{\prime \prime } - a^{2} \Theta } \right) - \frac{{iaR_{D} }}{2}\left( {\left( {x\Theta^{\prime } } \right)^{\prime } - a^{2} x\Theta } \right) \\ & \quad + \frac{{iaR_{S} }}{2}\left( {\left( {x\Theta } \right)^{\prime \prime } - \Gamma^{\prime } - a^{2} x\Theta } \right) = - iac\left( {\Theta^{\prime \prime } - a^{2} \Theta } \right), \\ \end{aligned}$$

17 $$\left( {\Phi^{\prime \prime \prime \prime } + a^{4} \Phi - 2a^{2} \Phi^{\prime \prime } } \right) + H\gamma \left( {\Theta^{\prime \prime } - a^{2} \Theta } \right) - H\gamma \left( {\Phi^{\prime \prime } - a^{2} \Phi } \right) = - ia\alpha c\left( {\Phi^{\prime \prime } - a^{2} \Phi } \right),$$

18 $$\frac{1}{Le}\left( {\Gamma^{\prime \prime \prime \prime } + a^{4} \Gamma - 2a^{2} \Gamma^{\prime \prime } } \right) - \frac{{iaR_{D} }}{2}\left( {\left( {x\Gamma } \right)^{\prime \prime } - \Theta^{\prime } - a^{2} x\Gamma } \right) + \frac{{iaR_{S} }}{2}\left( {\left( {x\Gamma^{\prime } } \right)^{\prime } - a^{2} x\Gamma } \right) = - iac\left( {\Gamma^{\prime \prime } - a^{2} \Gamma } \right).$$

We multiply Eq. (16) by $\gamma \,\overline{\Theta }$, Eq. (17) by $\overline{\Phi }$ and Eq. (18) by $\overline{\Gamma }$, where the bar over the symbol denotes complex conjugate. Then, we integrate over $- 1 \le x \le 1$ and add the resulting equations to get

19 $$\begin{aligned} & \gamma \int\limits_{ - 1}^{1} {\left( {\Theta^{\prime \prime \prime \prime } \overline{\Theta } + a^{4} \left| \Theta \right|^{2} - 2a^{2} \Theta^{\prime \prime } \overline{\Theta }} \right)dx} + H\gamma \int\limits_{ - 1}^{1} {\left( {\Phi^{\prime \prime } \overline{\Theta } - a^{2} \Phi \overline{\Theta }} \right)} dx - H\gamma \int\limits_{ - 1}^{1} {\left( {\Theta^{\prime \prime } \overline{\Theta } - a^{2} \left| \Theta \right|^{2} } \right)} dx \\ & \quad - \frac{{ia\gamma R_{D} }}{2}\int\limits_{ - 1}^{1} {\left[ {\left( {x\Theta^{\prime } } \right)^{\prime } \overline{\Theta } - a^{2} x\left| \Theta \right|^{2} } \right]dx} + \frac{{ia\gamma R_{S} }}{2}\int\limits_{ - 1}^{1} {\left[ {\left( {x\Theta } \right)^{\prime \prime } \overline{\Theta } - \Gamma^{\prime } \overline{\Theta } - a^{2} x\left| \Theta \right|^{2} } \right]dx} \\ & \quad + \int\limits_{ - 1}^{1} {\left( {\Phi^{\prime \prime \prime \prime } \overline{\Phi } + a^{4} \left| \Phi \right|^{2} - 2a^{2} \Phi^{\prime \prime } \overline{\Phi }} \right)dx} + H\gamma \int\limits_{ - 1}^{1} {\left( {\Theta^{\prime \prime } \overline{\Phi } - a^{2} \Theta \overline{\Phi }} \right)} \,dx - H\gamma \int\limits_{ - 1}^{1} {\left( {\Phi^{\prime \prime } \overline{\Phi } - a^{2} \left| \Phi \right|^{2} } \right)} dx \\ & \quad + \frac{1}{Le}\int\limits_{ - 1}^{1} {\left( {\Gamma^{\prime \prime \prime \prime } \overline{\Gamma } + a^{4} \left| \Gamma \right|^{2} - 2a^{2} \Gamma^{\prime \prime } \overline{\Gamma }} \right)dx} - \frac{{iaR_{D} }}{2}\int\limits_{ - 1}^{1} {\left[ {\left( {x\Gamma } \right)^{\prime \prime } \overline{\Gamma } - \Theta^{\prime } \overline{\Gamma } - a^{2} x\left| \Gamma \right|^{2} } \right]dx} \\ & \quad + \frac{{iaR_{S} }}{2}\int\limits_{ - 1}^{1} {\left[ {\left( {x\Gamma^{\prime } } \right)^{\prime } \overline{\Gamma } - a^{2} x\left| \Gamma \right|^{2} } \right]dx} = - iac\int\limits_{ - 1}^{1} {\left[ {\gamma \left( {\Theta^{\prime \prime } \overline{\Theta } - a^{2} \left| \Theta \right|^{2} } \right) + \alpha \left( {\Phi^{\prime \prime } \overline{\Phi } - a^{2} \left| \Phi \right|^{2} } \right) + \left( {\Gamma^{\prime \prime } \overline{\Gamma } - a^{2} \left| \Gamma \right|^{2} } \right)} \right]} dx. \\ \end{aligned}$$

We now manipulate Eq. (19) by performing the integration by parts and using the boundary conditions. This procedure gives

20 $$\begin{aligned} & \gamma \int\limits_{ - 1}^{1} {\left( {\left| {\Theta^{\prime \prime } } \right|^{2} + a^{4} \left| \Theta \right|^{2} + 2a^{2} \left| {\Theta^{\prime } } \right|^{2} } \right)dx} + H\gamma \int\limits_{ - 1}^{1} {\left( {\left| {\Theta^{\prime } - \Phi^{\prime } } \right|^{2} + a^{2} \left| {\Theta - \Phi } \right|^{2} } \right)} dx \\ & \quad + \frac{ia}{2}\left( {R_{D} - R_{S} } \right)\int\limits_{ - 1}^{1} {x\left[ {\gamma \left( {\left| {\Theta^{\prime } } \right|^{2} + a^{2} \left| \Theta \right|^{2} } \right) + \left( {\left| {\Gamma^{\prime } } \right|^{2} + a^{2} \left| \Gamma \right|^{2} } \right)} \right]dx} + \frac{{iaR_{D} }}{2}\int\limits_{ - 1}^{1} {\left( {\Theta^{\prime } - \Gamma^{\prime}} \right)\overline{\Gamma }dx} \\ & \quad - \frac{{ia\gamma R_{S} }}{2}\int\limits_{ - 1}^{1} {\left( {\Gamma^{\prime } - \Theta^{\prime } } \right)\overline{\Theta }dx} + \int\limits_{ - 1}^{1} {\left( {\left| {\Phi^{\prime \prime } } \right|^{2} + a^{4} \left| \Phi \right|^{2} + 2a^{2} \left| {\Phi^{\prime } } \right|^{2} } \right)dx} + \frac{1}{Le}\int\limits_{ - 1}^{1} {\left( {\left| {\Gamma^{\prime \prime } } \right|^{2} + a^{4} \left| \Gamma \right|^{2} + 2a^{2} \left| {\Gamma^{\prime } } \right|^{2} } \right)dx} \\ & \quad = iac\int\limits_{ - 1}^{1} {\left[ {\gamma \left( {\left| {\Theta^{\prime } } \right|^{2} + a^{2} \left| \Theta \right|^{2} } \right) + \alpha \left( {\left| {\Phi^{\prime } } \right|^{2} + a^{2} \left| \Phi \right|^{2} } \right) + \left( {\left| {\Gamma^{\prime } } \right|^{2} + a^{2} \left| \Gamma \right|^{2} } \right)} \right]} dx, \\ \end{aligned}$$

where

21 $$\left| {\Theta^{\prime } - \Phi^{\prime } } \right|^{2} = \left| {\Theta^{\prime } } \right|^{2} + \left| {\Phi^{\prime } } \right|^{2} - \Phi^{\prime } \overline{\Theta }^{\prime } - \Theta^{\prime } \overline{\Phi }^{\prime } ,\left| {\Theta - \Phi } \right|^{2} = \left| \Theta \right|^{2} + \left| \Phi \right|^{2} - \Phi \overline{\Theta } - \Theta \overline{\Phi }.$$

The fourth and fifth terms on the left-hand side of Eq. (20) discard the possibility of arriving at any definite conclusion in deciding the sign of $c_{i}$ as it may be positive or negative. This is another instance wherein the Gill–Rees method of proving the stability of fluid flow becomes ineffective. For a single component system ($R_{S} = 0$), however, the above equation simply reduces to

22 $$\begin{aligned} & \gamma \int\limits_{ - 1}^{1} {\left( {\left| {\Theta^{\prime \prime } } \right|^{2} + a^{4} \left| \Theta \right|^{2} + 2a^{2} \left| {\Theta^{\prime } } \right|^{2} } \right)dx} + H\gamma \int\limits_{ - 1}^{1} {\left( {\left| {\Theta^{\prime } - \Phi^{\prime } } \right|^{2} + a^{2} \left| {\Theta - \Phi } \right|^{2} } \right)} dx \\ & \quad + \frac{{ia\gamma R_{D} }}{2}\int\limits_{ - 1}^{1} {x\left( {\left| {\Theta^{\prime } } \right|^{2} + a^{2} \left| \Theta \right|^{2} } \right)dx} + \int\limits_{ - 1}^{1} {\left( {\left| {\Phi^{\prime \prime } } \right|^{2} + a^{4} \left| \Phi \right|^{2} + 2a^{2} \left| {\Phi^{\prime } } \right|^{2} } \right)dx} \\ & \quad \quad = iac\int\limits_{ - 1}^{1} {\left[ {\gamma \left( {\left| {\Theta^{\prime } } \right|^{2} + a^{2} \left| \Theta \right|^{2} } \right) + \alpha \left( {\left| {\Phi^{\prime } } \right|^{2} + a^{2} \left| \Phi \right|^{2} } \right)} \right]} dx. \\ \end{aligned}$$

Equating the real part on both sides of the above equation allows one to conclude that $c_{i}$ is always strictly negative for all infinitesimal perturbations. Hence, the basic state is asymptotically stable and this conclusion is in conformity with Rees's proof of stability^19^. The lack of formal proof of stability due to the presence of an additional diffusing component leaves an open possibility to investigate the stability or instability of the basic flow through a numerical solution.

## Numerical procedure

The Chebyshev collocation method is used to solve the eigenvalue problem constituted by Eqs. (11)–(15). The Chebyshev polynomial of *kth* order is given by

23 $$\xi_{k} (x) = \cos (k\cos^{ - 1} x),$$

with collocation points

24 $$x_{j} = \cos \left( {\frac{\pi j}{N}} \right),\quad j = 0,1,2, \ldots ,N,$$

where $N$ is the number of Chebyshev polynomials. Here, $j = 0$ and $N$ correspond to the right and left wall boundaries, respectively. The field variables $\Psi ,\,\Theta ,\,\Phi$ and $\Gamma$ are approximated in terms of the Chebyshev variable as follows

25 $$\Psi (x) = \sum\limits_{j = 0}^{N} {\Psi_{j} } \xi_{j} (x),\Theta (x) = \sum\limits_{j = 0}^{N} {\Theta_{j} } \xi_{j} (x),\Phi (x) = \sum\limits_{j = 0}^{N} {\Phi_{j} } \xi_{j} (x),\Gamma (x) = \sum\limits_{j = 0}^{N} {\Gamma_{j} } \xi_{j} (x),$$

where $\,\Psi_{j}$, $\Theta_{j}$, $\Phi_{j}$ and $\Gamma_{j}$ are constants. Equations (11)-(15) are discretized in terms of Chebyshev polynomials to get

26 $$\left( {\sum\limits_{k = 0}^{N} {B_{jk} \Psi_{k} } - a^{2} \Psi_{j} } \right) - R_{D} \sum\limits_{k = 0}^{N} {A_{jk} \Theta_{k} } + R_{S} \sum\limits_{k = 0}^{N} {A_{jk} \Gamma_{k} } = 0,$$

27 $$- \left( {{{ia} \mathord{\left/ {\vphantom {{ia} 2}} \right. \kern-\nulldelimiterspace} 2}} \right)\left\{ {\left( {R_{D} - R_{S} } \right)x_{j} \Theta_{j} - \Psi_{j} } \right\} + \left( {\sum\limits_{k = 0}^{N} {B_{jk} \Theta_{k} } - a^{2} \Theta_{j} } \right) + H(\Phi_{j} - \Theta_{j} ) = - iac\Theta_{j} ,$$

28 $$\left( {\sum\limits_{k = 0}^{N} {B_{jk} \Phi_{k} } - a^{2} \Phi_{j} } \right) - H\gamma (\Phi_{j} - \Theta_{j} ) = - ia\alpha c\Phi_{j} ,$$

29 $$- \left( {{{ia} \mathord{\left/ {\vphantom {{ia} 2}} \right. \kern-\nulldelimiterspace} 2}} \right)\left\{ {\left( {R_{D} - R_{S} } \right)x_{j} \Gamma_{j} - \Psi_{j} } \right\} + \frac{1}{Le}\left( {\sum\limits_{k = 0}^{N} {B_{jk} \Gamma_{k} } - a^{2} \Gamma_{j} } \right) = - iac\Gamma_{j} ,$$

30 $$\Psi_{0} = \Psi_{N} = 0,\,\Theta_{0} = \Theta_{N} = 0,\,\Phi_{0} = \Phi_{N} = 0,\,\Gamma_{0} = \Gamma_{N} = 0,$$

where

31 $$\begin{aligned} A_{00} & = \frac{{\left( {2N^{2} + 1} \right)}}{6} = - A_{NN} ,\,A_{jk} = \frac{{c_{j} ( - 1)^{k + j} }}{{c_{k} (x_{j} - x_{k} )}};\,j \ne k,A_{jk} = - \frac{{x_{j} }}{{2(1 - x_{j}^{2} )}};\,1 \le k = j \le N - 1, \\ B_{jk} & = A_{jm} \cdot A_{mk} , \\ \end{aligned}$$

with

32 $$c_{j} = \left\{ {\begin{array}{*{20}l} 1 \hfill & {1 \le j \le N - 1} \hfill \\ 2 \hfill & {j = 0,N} \hfill \\ \end{array} } \right..$$

Equations (26)–(30) lead to a generalized eigenvalue problem of the form

33 $$AX = cBX,$$

where $A$ and $B$ are square complex matrices of order $4\left( {N + 1} \right)$, $c$ is the eigenvalue and $X$ is the eigenfunction. For fixed values of governing parameters $R_{S} ,\,H,\,\gamma ,\alpha ,Le$ and $a$ the value of $c_{r}$ at which $c_{i} = 0$ is found by varying $R_{D}$, which is accomplished by the secant method for a fixed point determination. In accomplishing this we have selected the eigenvalue having the largest imaginary part. Repeating this procedure for different values of $a$, the marginal stability curve is obtained, say $R_{D}^{*} \left( {a,R_{S} ,\,H,\,\gamma ,\alpha ,Le} \right)$ and the corresponding frequency $c_{r}^{*} \left( {a,R_{S} ,\,H,\,\gamma ,\alpha ,Le} \right)$. The critical values $R_{Dc} \left( {a_{c} ,R_{S} ,\,H,\,\gamma ,\alpha ,Le} \right) = \mathop {\min }\limits_{a} R_{D}^{*} \left( {a,R_{S} ,\,H,\,\gamma ,\alpha ,Le} \right)$ and $c_{c} \left( {a_{c} ,R_{S} ,\,H,\,\gamma ,\alpha ,Le} \right)$ are then obtained for specified values of $R_{S} ,\,H,\,\gamma ,\alpha$ and $Le$^30,31^. If $c_{c} = 0$ then the critical disturbance modes are stationary otherwise they are travelling-waves. It should be noted that $R_{Dc} = \min \left( {R_{Dc}^{S} ,R_{Dc}^{T} } \right)$ if both the modes exist, where $R_{Dc}^{S}$ and $R_{Dc}^{T}$ are the critical thermal Darcy–Rayleigh numbers for the stationary and the travelling-wave modes, respectively.

The convergence of the numerical scheme used is tested by evaluating the response of the number of collocation points $N$ on the critical stability parameters for different sets of governing parameters. The computed values so obtained are tabulated in Table 1 and it is seen that the accuracy improves as $N$ increases. Regardless of the values of the governing parameters, the critical values for distinct values of $N$ exceeding 14 are identical up to 5 decimal places. Therefore, all of the computations are reported by taking $N = 15$.

**Table 1:** Convergence tests of the Chebyshev collocation method for representative values of governing parameters.

$N$	$R_{S} = 500,\,\gamma = 1,\,Le = 0.5,H = 1$			$R_{S} = 1000,\,\gamma = 1,\,Le = 0.5,H = 1$			$R_{S} = 500,\,\gamma = 1,\,Le = 0.5,H = 100$			$R_{S} = 500,\,\gamma = 10,\,Le = 0.5,H = 1$			$R_{S} = 500,\,\gamma = 1,\,Le = 10,H = 1$		
	$R_{Dc}$	$a_{c}$	$c_{c}$	$R_{Dc}$	$a_{c}$	$c_{c}$	$R_{Dc}$	$a_{c}$	$c_{c}$	$R_{Dc}$	$a_{c}$	$c_{c}$	$R_{Dc}$	$a_{c}$	$c_{c}$
3	455.15806	0.569400	0	904.16424	0.290263	0	458.54404	1.304487	± 9.624306	445.32753	0.449990	0	127.51952	2.236738	± 89.312561
4	416.98237	0.355783	0	829.30129	0.176603	0	487.79367	1.318743	± 9.639258	397.68067	0.286170	0	347.89680	0.279217	± 18.969205
6	413.72210	0.326146	0	824.03654	0.161680	0	477.92587	1.191875	± 10.015439	396.15973	0.263758	0	300.92250	1.057651	± 58.751588
8	413.59792	0.326841	0	823.73027	0.162086	0	478.10165	1.188263	± 9.954005	395.81154	0.264629	0	342.99234	1.226615	± 41.552617
10	413.56882	0.326854	0	823.67317	0.162092	0	478.12514	1.188081	± 9.952649	395.78540	0.264643	0	348.15457	1.715510	± 11.466295
11	413.57079	0.326850	0	823.67705	0.162091	0	478.12542	1.188079	± 9.952635	395.78690	0.264642	0	352.19606	2.164755	0
12	413.56977	0.326855	0	823.67495	0.162093	0	478.12567	1.188075	± 9.952608	395.78597	0.264645	0	359.16319	2.750712	± 9.071511
13	413.56964	0.326856	0	823.67470	0.162093	0	478.12576	1.188076	± 9.952607	395.78587	0.264646	0	352.87541	0.028388	0
14	413.56969	0.326856	0	823.67480	0.162093	0	478.12578	1.188076	± 9.952605	395.78592	0.264645	0	352.87541	0.028388	0
15	413.56969	0.326856	0	823.67480	0.162093	0	478.12578	1.188076	± 9.952605	395.78592	0.264645	0	352.87541	0.028388	0

## Results and discussion

The Gill–Rees stability problem is extended to capture the influence of a second diffusing component which has precluded the possibility of proving the stability of base flow analytically. The eigenvalue problem is solved numerically using the Chebyshev collocation method. The present problem contains six parameters such as the Darcy–Rayleigh number $R_{D}$, the solutal Darcy–Rayleigh number $R_{S}$, the Lewis number $Le$, the scaled interphase heat transfer coefficient *H*, the porosity modified thermal conductivities ratio $\gamma$ and the ratio of thermal diffusivities $\alpha$. The results are presented in terms of critical values of $R_{D}$ computed with respect to the wave number $a$ for various values of $R_{S}$, $Le$, *H* and $\gamma$ assuming $\alpha$ to be unity. To explore the several limiting cases, the parameters are considered in the ranges, $H \in \left[ {10^{ - 5} ,10^{4} } \right]$, $\gamma \in \left[ {10^{ - 2} ,10} \right]$, $R_{S} \in \left[ {0,2000} \right]$ and the Lewis number is chosen in the range 0.1–10^32^.

### Growth rate analysis

**Figure 2:**
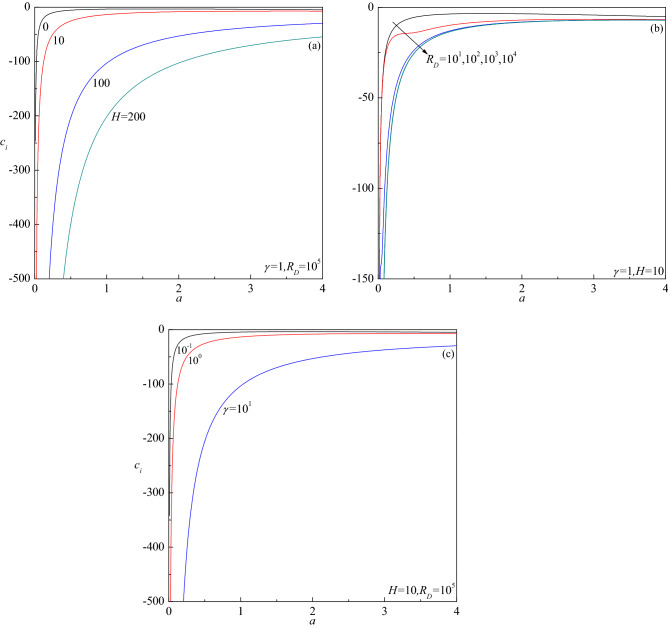
Growth rate $c_{i}$ versus $a$ for different values of (**a**) $H$, (**b**) $R_{D}$ and (**c**) $Le$, in the absence of a second-diffusing component ($R_{S} = 0$).

**Figure 3:**
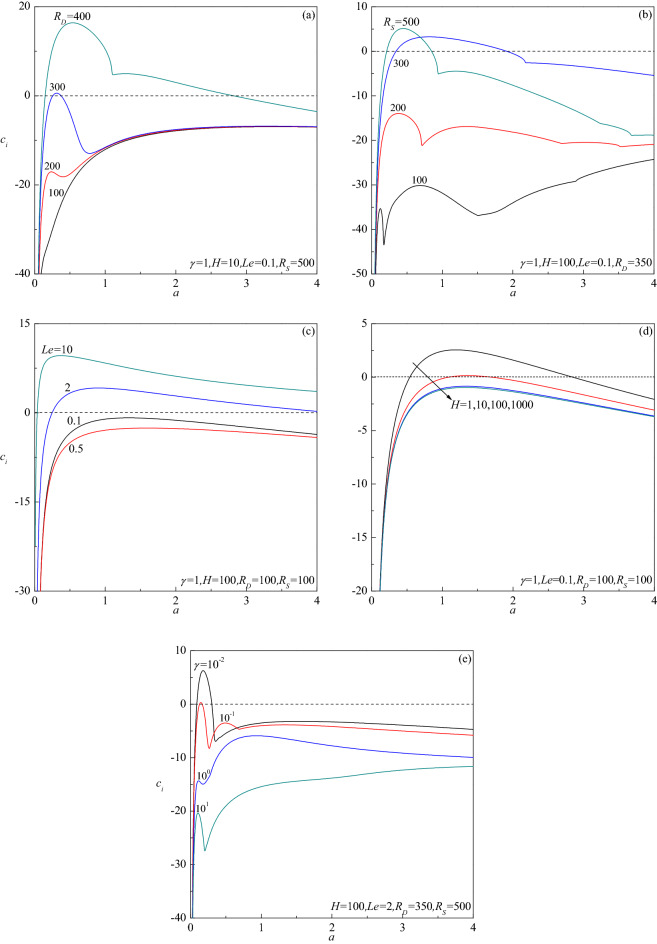
Growth rate $c_{i}$ versus $a$ for different values of (**a**) $R_{D}$, (**b**) $R_{S}$, (**c**) $Le$, (**d**) $H$ and (**e**) $\gamma$.

The growth rate of normal modes is pursued by computing the complex eigenvalue $c_{r} + ic_{i}$ for the assigned governing parameters. The most essential information regarding the stable/unstable behavior of the basic flow comes from the sign of the growth rate $c_{i}$. Plots of $c_{i}$ versus the wave number $a$ for different values of $H$, $R_{D}$ and $\gamma$ when $R_{S} = 0$ are reported in Fig. 2a–c. The numerical data reported in these figures support the conclusion that $c_{i}$ is negative in every case suggesting that no instability is possible. This result agrees with the analytical proof presented by Rees^19^. Nonetheless, the results portrayed for $R_{S} \ne 0$ in Fig. 3a–e demonstrate the possibility of $c_{i}$ undergoing a transition from negative to positive with increasing $a$. This indicates the chances of base flow becoming unstable in the presence of a second diffusing component depending on the values of governing parameters. A closer look at these figures also reveals that larger values of $R_{D}$ (Fig. 3a), $R_{S}$ (Fig. 3b) and $Le$ (Fig. 3c), while smaller values of $H$(Fig. 3d) and $\gamma$ (Fig. 3e) are necessary for $c_{i}$ to be positive.

### Neutral stability curves

**Figure 4:**
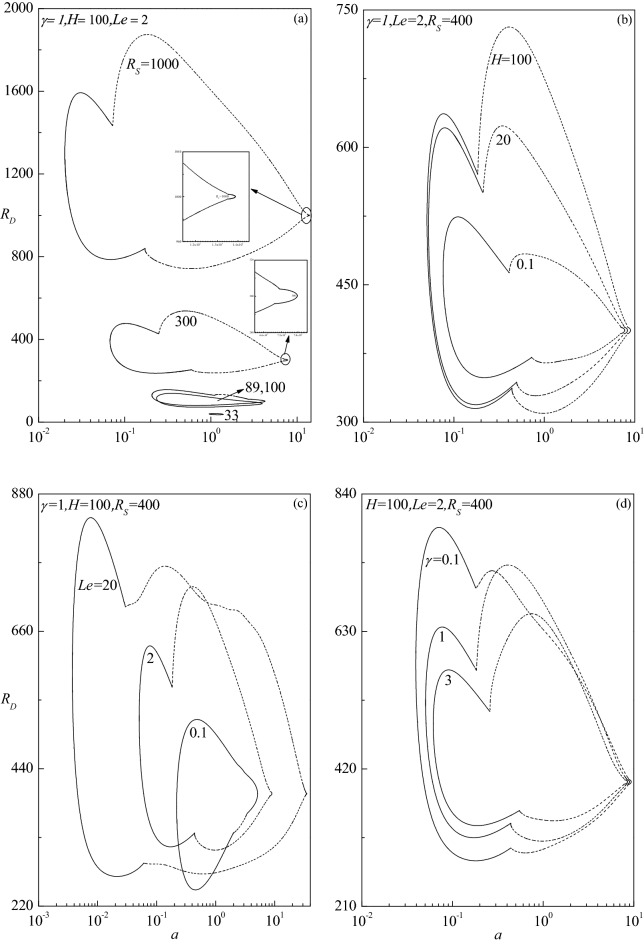
Evolution of neutral stability curves in the $(a,R_{D} )$-plane for different values of (**a**) $R_{S}$, (**b**) $H$, (**c**) $Le$ and (**d**) $\gamma$. The solid and dashed lines respectively denote the stationary and travelling-wave modes. This convention applies to all the figures hereafter.

A systematic study on the topology of neutral stability curves is carried out. The neutral stability curve which delimits the boundary between the regions of parametric stability and instability is generated by specifying a vanishing growth rate $(c_{i} = 0)$ of the perturbation modes. Figure 4a–d display the neutral stability curves for different values of $R_{S}$, $H$, $Le$ and $\gamma$. Some novel consequences not perceived either in double-diffusive vertical porous (LTE model) or non-porous domains are found for certain choices of parametric values. The neutral stability curves form a loop comprising both stationary and travelling-wave modes, in some cases, exhibiting a local minimum of their own and the least among the two determines the nature of instability mode. The region inside the loop corresponds to instability ($c_{i} > 0$) and the outside defines the parametric conditions of linear stability $(c_{i} < 0)$. This indicates the requirement of two values of the thermal Darcy–Rayleigh number to specify the linear instability criteria.

Figure 4a shows the evolution of neutral stability curves for different values of $R_{S}$ when $\gamma = 1,\,\,Le = 2$ and $H = 100$. For $R_{S} =$ 1000, it is seen that the closed neutral curve consists of travelling-wave mode, which is connected on either side by those of stationary mode; an unusual phenomenon not observed in the earlier studies of double-diffusive convection^28,33^. While both the modes exhibit local minimum, the least among the two corresponds to the travelling-wave mode which dominates the mode of instability. Although a similar trend exists for $R_{S} = 300$, the instability region gets reduced and the mode of instability turns out to be stationary in this case. At $R_{S} = 100$, the instability region gets further diminished. Moreover, the neutral curve of travelling-wave mode appears only in the upper portion of the loop confining to a smaller range of wave number $a$ and the preferred mode of instability remains to be stationary. The region of instability continues to diminish for $R_{S}$ = 89 and 33 and now the closed loop consists only of the neutral curve of stationary mode. The region of instability eventually disappears with a further decrease in the value of $R_{S}$ because the perturbations exhibit a negative growth rate.

**Figure 5:**
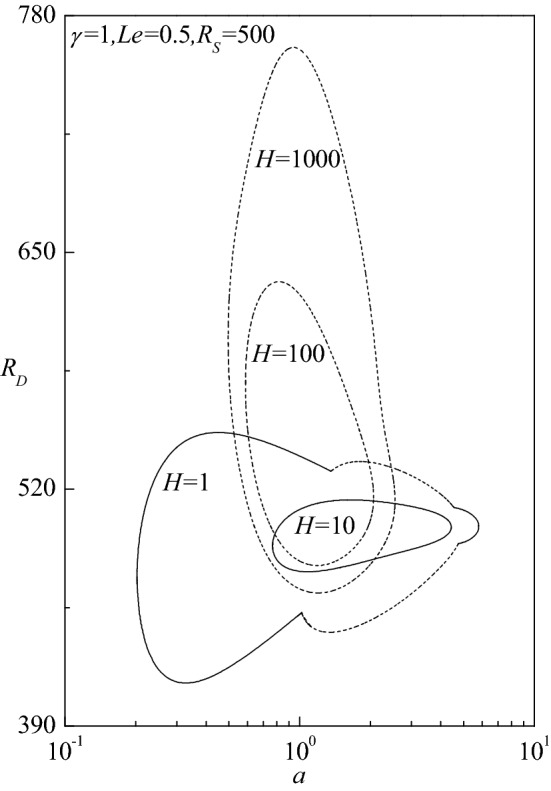
Evolution of neutral stability curves in the $(a,R_{D} )$-plane for different values of $H$**.**

Figure 4b–d exhibit the way in which the neutral curves evolve for different values of $H$ (with $\gamma = 1,\,Le = 2,R_{S} = 400$), $Le$ (with $\gamma = 1,\,H = 100,\,R_{S} = 400$) and $\gamma$ (with $H = 100,\,Le = 2,R_{S} = 400$), respectively. The outline of neutral curves is akin to those identified in Fig. 4a. It is seen that the effect of increasing $H$ and $Le$ as well as decreasing $\gamma$ is to enlarge the size of the instability region. For $H$ = 0.1 and 20, the instability occurs through the stationary mode, while for $H$ = 100 it switches over to the travelling-wave mode (Fig. 4b). The pattern of instability keeps shifting between the stationary and travelling-wave modes with increasing $Le$ (Fig. 4c) and $\gamma$ (Fig. 4d). The sensitivity of governing parameters on the progression of neutral curves is examined and displayed in Fig. 5 for various values of $H$ when $\gamma = 1,\,Le = 0.5$ and $R_{S} = 500$. The neutral curve loop includes both stationary and travelling-wave modes for $H = 1$ as noted earlier, while for $H$ = 10 it consists of only stationary mode but for $H = 100$ and 1000 it consists of only travelling-wave mode. Thus, increase in $H$ is to alter the mode of instability from the stationary to the travelling-wave mode. In addition, the instability region corresponding to the travelling-wave mode gets elongated vertically with increasing $H$.

### Stability boundaries

**Figure 6:**
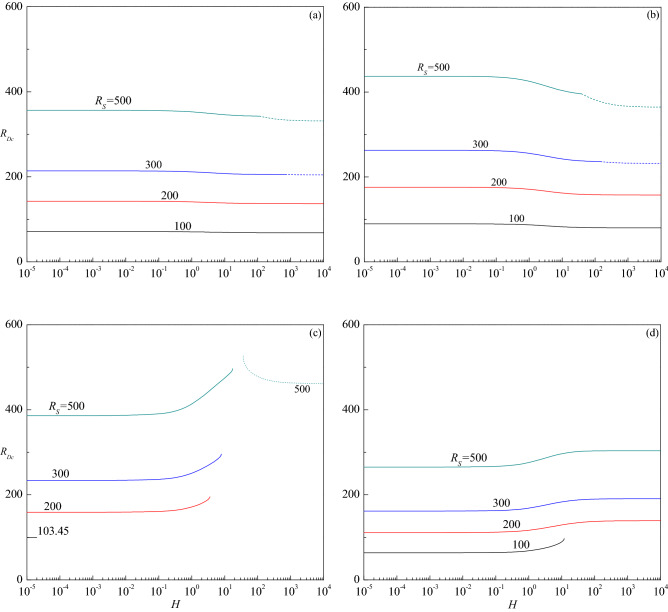
The variation of $R_{Dc}$ versus $H$ for different values of $R_{S}$ relative to the cases (**a**) $Le = 10$, (**b**) $Le = 2$, (**c**) $Le = 0.5$ and (**d**) $Le = 0.1$, when $\gamma = 1$.

The critical thermal Darcy–Rayleigh number $R_{Dc}$, the corresponding critical wave number $a_{c}$ and the critical wave speed $c_{c}$, are computed for different values of governing parameters to know their impact on the stability of fluid flow. The trend of $R_{Dc}$ with $H$ for different values of $R_{S}$ is shown in Fig. 6a–d for $Le = 10,$ 2, 0.5 and 0.1, respectively. These figures show that the effect of increasing $R_{S}$ is to increase $R_{Dc}$ and stabilize the fluid flow. The critical thermal Darcy–Rayleigh number, existing for those values of $R_{S}$ and $Le$ throughout the range of $H$, is found to be invariant and approaches different limits at the extreme values of $H < < 1$ and $H > > 1$. It is worth mentioning here that the definition of thermal Darcy–Rayleigh number is not the one usually employed when dealing with the LTE situation ($H \to \infty$). In this limit, the LTE definition of thermal Darcy–Rayleigh number is $\tilde{R}_{D} = \gamma R_{D} /(1 + \gamma )$. The limit $H \to 0$ yields a regime utterly opposite to that of LTE due to the temperature fields of solid and fluid phases being completely independent of this limit. In fact, in the limit of vanishingly small $H$, the critical thermal Darcy–Rayleigh number attains the lowest (highest) possible values for the onset of instability when the Lewis number $Le < 1$($Le > 1$). Though Fig. 6a, b exhibit the instability to initiate as the stationary mode for $R_{S} = 300$ and 500, it switches over to the travelling-wave mode as the value of $H$ reaches some transition value $H_{T}$, which gets decreased with increasing $R_{S}$. To the contrary, the instability occurs only through the stationary mode for other values of $R_{S} ( = 100,200)$ throughout the range of $H$. Figure 6c shows an altogether different behavior. For each value of $R_{S}$ considered, the $R_{Dc}$ curves of the stationary mode end at some value of $H$ indicating the base flow is asymptotically stable thereafter but with an exception when $R_{S} = 500$ in which case the instability ensues again via travelling-wave mode after a certain value of $H$. In other words, there exists a finite range of $H$ in which the flow will be stable for $R_{S} = 500$. Another point to be noted from this figure is that the flow remains stable for $R_{S} < 103.45$ and becomes unstable for $R_{S} \ge 103.45$. This shows that instability exists for a certain parametric space of $R_{S}$ which prominently depends on $Le$ as observed in the LTE case^28^. Figure 6d exemplifies that the instability appears only in the form of stationary mode for all values of $R_{S}$ and $H$ considered, while for $R_{S} = 100$ the curve ceases at some value of $H$ as observed in Fig. 6c. From the figures, it is also obvious that the effect of increasing $Le$(> 1) is to hasten and on the contrary increase in $Le$(< 1) is to delay the onset of instability for a fixed value of $R_{S}$. One common feature that emanates from these outcomes is that there exists a threshold value of $R_{S}$ exceeding which only the base flow becomes unstable and below which the flow remains to be stable as observed in the single diffusive component case^19^. The threshold value of $R_{S}$ is found to depend strongly on the values of other governing parameters. Alternatively, we also considered the plots of critical solute Darcy–Rayleigh number $R_{Sc}$ versus $H$ for different values of $R_{D}$ for a fixed value of $Le$ (Figures are not shown) to get an insight into the problem. We witnessed that the overall behavior perceived was akin to that of Fig. 6, except the difference in the values of $R_{Sc}$ and $R_{Dc}$.

**Figure 7:**
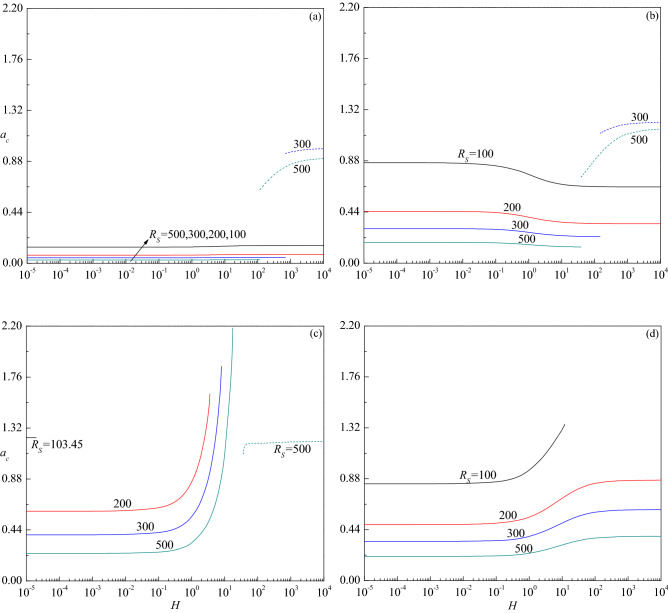
The variation of $a_{c}$ versus $H$ for different values of $R_{S}$ relative to the cases (**a**) $Le = 10$, (**b**) $Le = 2$, (**c**) $Le = 0.5$ and (**d**) $Le = 0.1$, when $\gamma = 1$.

**Figure 8:**
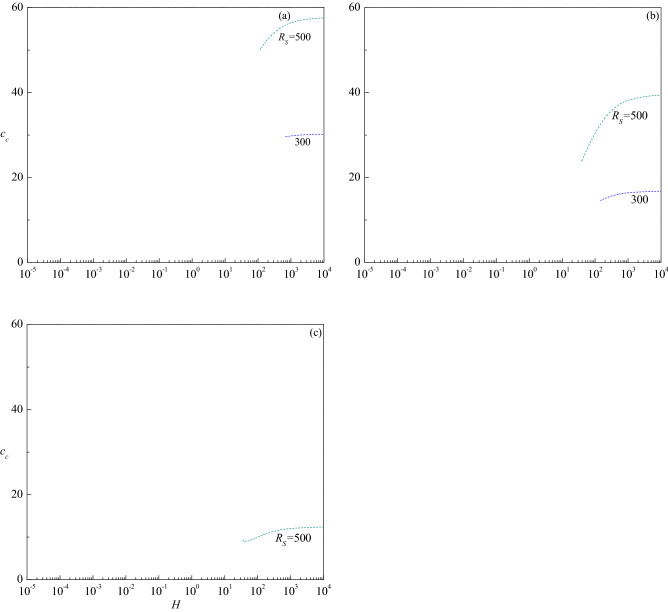
The variation of $c_{c}$ versus $H$ for different values of $R_{S}$ relative to the cases (**a**) $Le = 10$, (**b**) $Le = 2$ and (**c**) $Le = 0.5$ when $\gamma = 1$.

The variation of critical wave number $a_{c}$ with $H$ is presented in Fig. 7a–d for the corresponding parametric values considered in Fig. 6a–d. The figures show that increasing $R_{S}$ is to decrease $a_{c}$ in both stationary and travelling-wave modes for all values of $Le$ considered and hence its effect is to enlarge the size of convection cells. Even though the critical wave number of stationary mode increases with $H$ for $Le = 10$, the insignificant variation in its value is apparent from Fig. 7a. However, $a_{c}$ of travelling-wave mode instability for $R_{S}$ = 300 and 500 initially increases slightly and remains to be constant with $H$. An opposite trend follows for $Le = 2$ and the changes in $a_{c}$ with $H$ are somewhat noticeable in the stationary mode (Fig. 7b). Figure 7c displays the results for $Le = 0.5$ and observes a steep increase in $a_{c}$ of the stationary mode with increasing $H$ except for $R_{S}$ = 103.45. However, $a_{c}$ increases slightly before attaining a constant value at higher values of $H$ when the instability changes to the travelling-wave mode for $R_{S}$ = 500. The plots of $a_{c}$ for $Le = 0.1$ increase at the intermediate values of $H$ for all values of $R_{S}$ but end at some value of $H$ for $R_{S}$ = 100 (Fig. 7d). The figures further disclose that the critical wave number decreases (increases) with increasing $Le$ > 1 ($Le$ < 1) for each value of $R_{S}$. Figure 8a–c show the corresponding change of critical wave speed $c_{c}$ as a function of $H$ for $Le = 10$, 2 and 0.5, respectively since the instability occurs through the stationary mode for $Le = 0.1$. These figures show an interruption in the curves of $c_{c}$ with $H$ due to a change in the mode of instability from the stationary mode $\left( {c_{c} = 0} \right)$ to the traveling-wave mode $\left( {c_{c} \ne 0} \right)$. It is also noted that $c_{c}$ increases with increasing $R_{S}$, $H$ and $Le$, and attains a constant value as $H \to \infty$.

**Figure 9:**
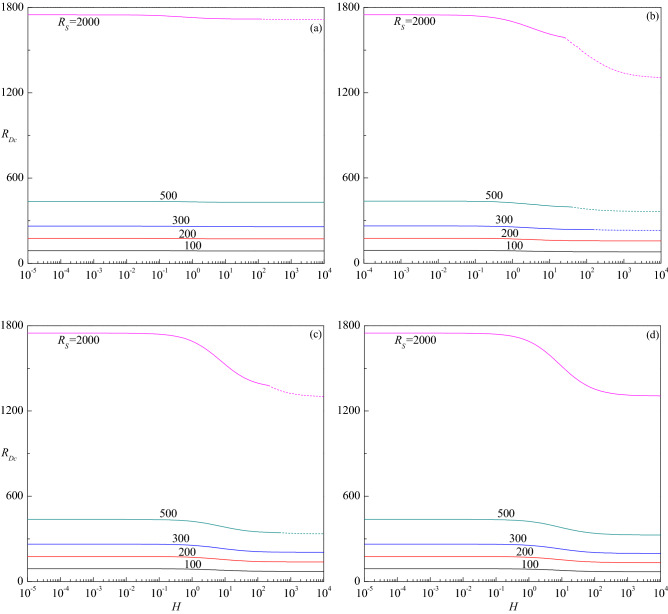
The variation of $R_{Dc}$ versus $H$ for different values of $R_{S}$ relative to the cases (**a**) $\gamma = 10$, (**b**) $\gamma = 1$, (**c**) $\gamma = 0.1$ and (**d**) $\gamma = 0.01$ when $Le = 2$.

**Figure 10:**
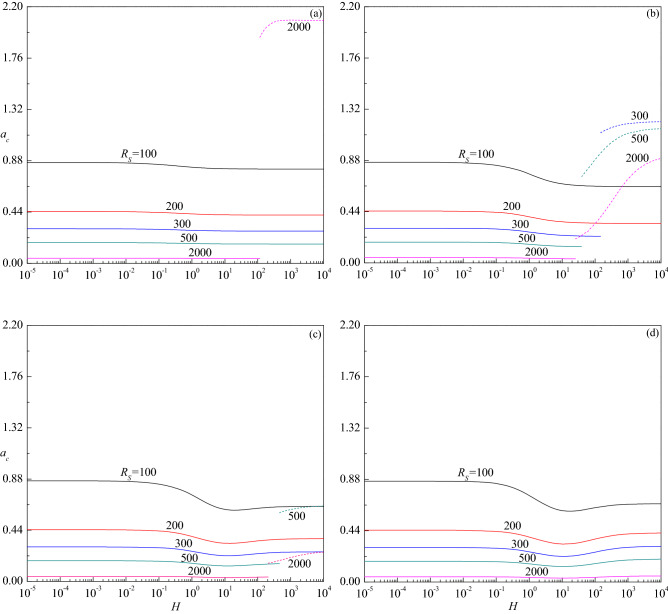
The variation of $a_{c}$ versus $H$ for different values of $R_{S}$ relative to the cases (**a**) $\gamma = 10$, (**b**) $\gamma = 1$, (**c**) $\gamma = 0.1$ and (**d**) $\gamma = 0.01$ when $Le = 2$.

**Figure 11:**
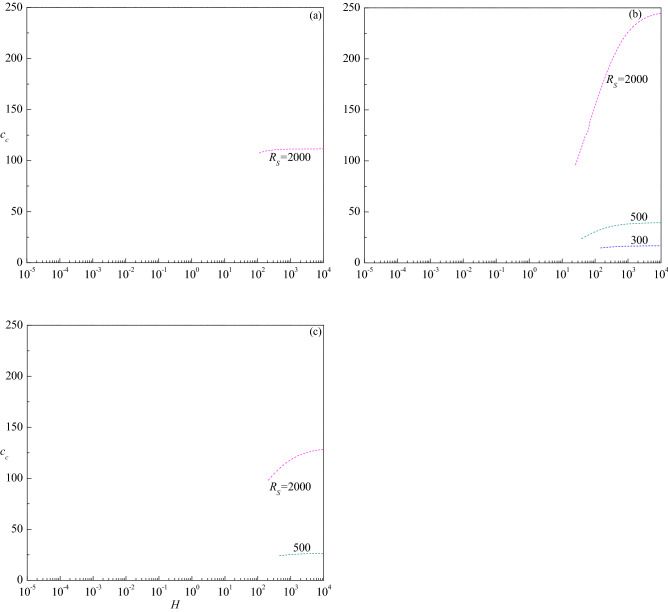
The variation of $c_{c}$ versus $H$ for different values of $R_{S}$ relative to the cases (**a**) $\gamma = 10$, (**b**) $\gamma = 1$ and (**c**) $\gamma = 0.1$ when $Le = 2$.

Figure 9a–d demonstrate the impact of porosity modified conductivities ratio $\gamma$ on the variation of $R_{Dc}$ as a function of $H$ for different values of $R_{S}$. For $\gamma$ = 0.01, it is observed that the instability sets in always via stationary mode throughout the range of $H$ for the values of $R_{S}$ considered (Fig. 9d). However, depending on the value of $R_{S}$, the shifting of instability from the stationary to the travelling-wave mode emerges after certain values of $H$ for $\gamma$ = 0.1 (Fig. 9c), 1 (Fig. 9b) and 10 (Fig. 9a). Despite the critical thermal Darcy–Rayleigh number assuming different values at extreme values of $H \to 0$ and $H \to \infty$ for smaller and moderate values of $\gamma$ (Fig. 9b–d), it becomes independent of $H$ at sufficiently large values of $\gamma$ (Fig. 9a). This is because, $\tilde{R}_{D} = R_{D}$ as $\gamma \to \infty$ and this describes a fluid with an extremely high thermal conductivity. This explains why the temperature of the fluid is not influenced by that of the solid, while the temperature of the solid is locally coincident with that of the fluid. As a result, interphase convection is prevented at the pore level, but extremely efficient heat conduction in the fluid creates a perfect thermal link between the phases. Moreover, both $R_{Dc}$ and $a_{c}$ become independent of $\gamma$ at lower values of $H$ as the solid phase does not affect the onset criterion. On the other hand, $R_{Dc}$ and $a_{c}$ varies significantly with $\gamma$ at higher values of *H* since the stability criterion depends on the mean properties of the medium. Also, an increase in the value of $\gamma$ is to increase $R_{Dc}$ indicating its effect is to stabilize the fluid flow (Fig. 9a–d). Whereas, the critical wave number increases with increasing $\gamma$ at the travelling-wave mode while a mixed behavior could be seen at the stationary mode (Fig. 10a–d). The above observed phenomena are true for all the considered values of $R_{S}$. The critical wave speed shows both increasing and decreasing trends with increasing $\gamma$ (Fig. 11a–d).

## Conclusions

The implication of a solute concentration field on the stability of thermal convection in a vertical porous layer subject to different constant temperatures and solute concentrations at the impermeable boundaries is investigated. The Darcy law has been employed in a framework based on the Oberbeck–Boussinesq approximation and the LTNE model has been exploited by assuming two different local temperatures for the fluid and solid phases of the porous medium. To study the stability of the basic flow, a linear stability analysis has been performed by employing a normal mode analysis of the eigenvalue problem. It is observed that the Gill–Rees proof turns out to be ineffective in deciding the stability of the base flow. A numerical analysis has been carried out to extract the critical value of the thermal Darcy–Rayleigh number $R_{D}$ that identifies the threshold for the onset of instability. It has been established that the second diffusing component evidences a dramatic effect on the stability of the basic flow. A systematic study on the topology of neutral stability curves has been carried out and some remarkable departures from those of LTE regime are observed.

The principal results of the foregoing linear stability analysis can be outlined as follows:

- In contrast to the Gill–Rees stability problem, the presence of a solute concentration field initiates the possibility of base flow becoming unstable under certain parametric conditions.
- Closed neutral stability curves comprising stationary and/or travelling-wave modes exist depending on the choices of governing parameters indicating the requirement of two thermal Darcy–Rayleigh numbers to specify the linear instability criteria instead of the usual single value.
- The mode of instability switches over from the stationary mode to the travelling-wave mode as the local minimum of the neutral stability curve loop formed by these two modes gets interchanged for certain choices of parameters. In particular, the changing over of the preferred mode of instability depends prominently on the value of the scaled interphase heat transfer coefficient $H$ which increases with the decrease in the solutal Darcy–Rayleigh number $R_{S}$ and an increase in the Lewis number $Le$. However, a mixed behavior is noticed with an increase in the ratio of porosity-modified thermal conductivities $\gamma$.
- The instability of fluid flow is independent of $H$ at its extreme values for all the governing parameters. While the departure from the LTE regime leads to stabilization of the base flow when $Le > 1$ (i.e. thermal diffusivity of the fluid is greater than the thermal diffusivity of the solid), the trend gets reversed when $Le < 1$.
- Increase in $R_{S}$, $\gamma$ and $Le$(< 1) is to abet the stability of fluid flow and exactly an opposite behavior manifests with increasing $Le$(> 1).
- The size of the convection cells increases with increasing $R_{S}$ and $Le( > 1)$, while it decreases with increasing $Le( < 1)$. A mixed behavior is noticed with increasing $\gamma$.

We have explored the essence of thermosolutal natural convection through linear stability analysis in a broader context but it also requires further investigation through a weakly nonlinear stability analysis and direct numerical simulation. These analyses give more details about the flow instabilities beyond the small-amplitude stage. Also, one may consider the implications of partially permeable boundary conditions. With such a model, one can investigate the gradual transition from permeable to perfectly impermeable boundaries, and its effects on the onset conditions for the instability. These are left for future study.

## Data Availability

The data that supports the findings of this study are available within the article.
